# Words to Young Homœopaths

**Published:** 1887-04

**Authors:** D. C. McLaren

**Affiliations:** Brantford, Ontario


### WORDS TO YOUNG HOMŒOPATHS.

D. C. McLaren, M. D., Brantford, Ontario.

As yet a young homœopath myself, and still encountering difficulties, I feel privileged to speak to some who, perhaps, like myself, have found themselves, not long after leaving college, muddled and bewildered where they should have been easily successful. Most of us are taught very thoroughly by experience, but some never, and for the reason that elementary premises are wanting on which to base definite conclusions.

To illustrate, allopathic text-books on surgery, after giving the method of operation for fistula in ano, frequently conclude with some such wisdom as this: " Keep up the patient's strength and guard against lung disease." It seems never to have occurred to these possessors of all knowledge that the operation of slitting a fistula might have anything to do with producing the subsequent phthisis.

Among professing homœopaths similar instances, differing only in degree of crudity, come to one's knowledge almost every day. One has exercised his art to the best of his knowledge, and perhaps from want of confidence or want of patience to wait for results, rushes to allopathic palliatives. This, to be plain, is a species of imbecility, and such imbeciles will never succeed in practicing Homœopathy.

Another fails to cure promptly and yet hangs on to the case, bound to find the right remedy or to know the reason why. Of such a one we may well be hopeful. We cannot all be Hahnemanns or Herings, and yet we can all possess their secret. They were determined to know the reason of things, and, though it took a long lifetime, both succeeded. Failures we all have and must have, successes too, but we must learn most from our mistakes, otherwise we will go on repeating them year by year, even as do the allopaths. Having profited by failure, we will be in a fitter mood to profit by success more difficult in a sense than the other, because the exact elements of success in a given case are often harder to determine accurately than the causes of failure in another. Then, too, much of success that we have is only partial—not what it should be, not up to the mark, in fact. I remember well in my first year's practice sending the report of a case that I prided myself somewhat upon to one of our journals. The editor was kind enough to encourage me by printing it, but wrote that the case should have been cured in one week instead of three. As he showed me what was wrong and why, I learned something.

There are many pitfalls which, I suppose, only experience can teach us to avoid, and this only as our judgment becomes keener and more evenly balanced. Then, too, year by year we lose (or ought to lose, for it dies hard) that impatience of youth, which often interferes so disastrously.

I presume that the young practitioner is earliest liable to failure from what I may call "overdoing" it. He is summoned to acute and apparently alarming cases calling for decisive treatment; the symptoms are plain enough, and he leaves a supply of the indicated remedy in a glass of water. He may or he may not have been informed, what Hahnemann stated once half a century ago, that a single dose is sufficient to cure ordinary acute attacks, but hearing such a thing suffices little, for he has never seen that precept carried out, or, even if he has, the old popular feeling in favor of dosing may unconsciously urge him to do as we have supposed him doing. The next day, to his surprise, the patient is worse, and perhaps has some new symptoms; there is, surely, no doubt about it; some other remedy is required and given. In a day or two the case is inextricably muddled, and our tyro falls back on allopathy, or else the patient does the same thing by changing his physician.

The first case of acute rheumatism the writer ever had was in this wise. A workingman of advanced years was taken at the beginning of winter with pains, stiffness, and swelling in one foot. On inquiry he was found to be quite restless, the foot being easy in one position but for a few minutes, when a change was necessary. I gave him *Rhus tox.*, and left him with the assurance that he would be back at work in a few days. Instead of that, he was confined to his room all winter, and it was well on toward summer before he was fairly at work again. Why? Briefly, then, the second day found him, as he said, worse, or, as I perceived, different. Now he could not move at all, as every attempt was painful, and he felt better so long as he kept perfectly quiet. Wonder, surprise, and over-anxiety to keep my promise as to his recovery prevailed over cool judgment, and, in a sort of wild panic, I prescribed *Bryonia.*

Fortunately for me, the man was too poor to summon another physician, so I had six months' experience of the treatment of rheumatism at his expense, and a weary round of prescriptions it was. I learned enough, however, to be able to knock his next attack on the head in a few days, but not enough to prevent my muddling the case of a poor widow with a large family the following winter. From these cases, then, I learned definitely the following rules:

1. *Prescribe accurately.* If this is not possible at the bedside, leave some *placebo,* go home and study. Make sure of the remedy at all costs, then, if it be a lingering complaint like rheumatism, or one of definite days of progress, like pneumonia and typhoid when fully developed, give a single dose dry or in water spread over an hour or two. But if it be severe colic, gall stone, neuralgia, convulsions, etc., leave a supply in water, to take a dose at every spasm of pain, or after every stool or emesis, as the case may be, strictly enjoining that none be given after improvement commences. In this way I cannot recall one case that required more than a second, or at most a third, dose of medicine. Suppose now a case of gall-stone colic. It would at first sight seem heartless to leave the patient for an hour or two while one is studying up the remedy, but when one has seen such cases suffering for days at a time in spite of all the palliatives the regulars could bring to bear, including Morphia, sweet oil, and hot baths, and, on the other hand, has seen such a sufferer go quietly to sleep a few minutes after taking a dose of the homœopathic remedy, the choice is certainly in favor of the latter by long odds. Add this consideration, and the course I recommended will be seen to be the only true, the only safe, one. Nearly all this class of cases have a tendency to recur again and again for years under allopathic treatment, but one attack cured homœopathically often prevents any more for life, or, at most, one or two light attacks follow after a few months.

2. Let the medicine have time to do its work. Some one asks, how long would you wait? In answering such a question it must be taken for granted that an absolutely correct prescription has been made in the first place, for if not, the sooner that is done the better. Then again, one must be able to depend on the potency, and for this reason I would advise every one to start with grafts of the polychrests obtained from their preceptors or other physicians who have tried them well. *Remember that the length of time a remedy takes to act is no proof that it*

*is not the right one.* In many cases, both acute and chronic, repeating the dose will spoil the case and even kill the patient. Take, for instance, the two following cases: Childbed hemorrhage with retained placenta, fainting and gasping, gagging as if about to throw up. The remedy was repeated every five minutes, and for a time—it seemed fully half an hour—the patient grew worse; a change of remedy would probably have been fatal, but in a moment the placenta was expelled, the flow ceased, and the woman began to breathe more naturally and made a good recovery. Then again, an infant with bronchitis, labored breathing, quiet, indifferent, too weak to cry, and the nostrils waving with each respiratory effort; a single dose of the indicated remedy was given and the case watched with fear and trembling for *two days* before any improvement was manifest. The conclusion was inevitable that a second dose or a change of remedy would have been fatal. Now, in the cases called chronic, how long should one wait? The answer is plain and simple, wait as long as the improvement lasts. But suppose there is no improvement in a week? Well, wait two, three, or even four weeks. It is the general experience that good results fail to appear for many days. The patient will say in a week's time that he is no better. If the correct remedy has been given any interference will irretrievably spoil the case, while if time be given, the original dose often effects a complete cure. Every homœopath of any education can prescribe with fair accuracy for cases of chronic disease, but many still fail to cure simply from ignorance of the proper management of remedies. But where is all this to be learned? says one. In Hahnemann's *Organon*, a book not for reading, but hard study, and in his *Chronic Diseases*, unfortunately out of print, but the reprinting of which would surely pay as well if not better than some of the works that are coming out so thickly, and would certainly be of greatly more value.

[TO BE CONTINUED.]

